# Regulatory subunits of PKA define an axis of cellular proliferation/differentiation in ovarian cancer cells

**DOI:** 10.1186/1755-8794-1-43

**Published:** 2008-09-26

**Authors:** Chris Cheadle, Maria Nesterova, Tonya Watkins, Kathleen C Barnes, John C Hall, Antony Rosen, Kevin G Becker, Yoon S Cho-Chung

**Affiliations:** 1 Cellular Biochemistry Section, National Cancer Institute, Bethesda, USA; 2 Lowe Family Genomics Core, Johns Hopkins University, Baltimore, USA; 3 Division of Allergy and Clinical Immunology, Johns Hopkins University, Baltimore, USA; 4 Division of Rheumatology, The Johns Hopkins University, Baltimore, USA; 5 Gene Expression and Genomics Unit, National Institute on Aging, Baltimore, USA

## Abstract

**Background:**

The regulatory subunit of cAMP-dependent protein kinase (PKA) exists in two isoforms, RI and RII, which distinguish the PKA isozymes, type I (PKA-I) and type II (PKA-II). Evidence obtained from a variety of different experimental approaches has shown that the relative levels of type I and type II PKA in cells can play a major role in determining the balance between cell growth and differentiation. In order to characterize the effect of PKA type I and type II regulatory subunits on gene transcription at a global level, the PKA regulatory subunit genes for RIα and RIIβ were stably transfected into cells of the ovarian cancer cell line (OVCAR8).

**Results:**

RIα transfected cells exhibit hyper-proliferative growth and RIIβ transfected cells revert to a relatively quiescent state. Profiling by microarray revealed equally profound changes in gene expression between RIα, RIIβ, and parental OVCAR cells. Genes specifically up-regulated in RIα cells were highly enriched for pathways involved in cell growth while genes up-regulated in RIIβ cells were enriched for pathways involved in differentiation. A large group of genes (~3600) was regulated along an axis of proliferation/differentiation between RIα, parental, and RIIβ cells. RIα/wt and RIIβ/wt gene regulation was shown by two separate and distinct gene set analytical methods to be strongly cross-correlated with a generic model of cellular differentiation.

**Conclusion:**

Overexpression of PKA regulatory subunits in an ovarian cancer cell line dramatically influences the cell phenotype. The proliferation phenotype is strongly correlated with recently identified clinical biomarkers predictive of poor prognosis in ovarian cancer suggesting a possible pivotal role for PKA regulation in disease progression.

## Background

The critical role of cAMP acting as a second messenger and exerting control over the regulation of cell growth and differentiation in a wide variety of cell types has been well established [1-3]. Experimental evidence has shown that the selective modulation of two isoforms of cAMP-dependent protein kinase (PKA-I and PKA-II) act as positive and negative intracellular regulators [4], respectively, of cell growth. PKA-I is only transiently overexpressed in normal cells in response to the physiologic stimuli of cell proliferation while, in contrast, it is constitutively overexpressed in cancer cells and this over-expression is associated with poor prognosis in many different human cancers. The disruption of the normal balance between PKA isozymes is highly associated with tumorigenesis and tumor growth.

Global gene expression profiling by microarray has demonstrated that antisense suppression of RIα in PC3M prostate and LS-174T colon carcinoma cells, exogenously treated with RIα antisense oligonucleotides, simultaneously up-regulates RIIβ and down-regulates a wide range of genes involved in cell proliferation and transformation [5]. Conversely, the vector-mediated overexpression of RIIβ exhibits induction of differentiation genes along with the suppression of cell proliferation and can lead to a reversion of tumor phenotype. Thus, switching of PKA isozymes can cause tumor cells to undergo a phenotypic reversion of malignancy.

To investigate the molecular mechanisms of this phenomenon, ovarian carcinoma cells (OVCAR-8) were transfected with human genes encoding PKA RIα or RIIβ subunits. Ovarian cancer cells were chosen as a useful model system since cAMP-signaling has already been shown to be of vital importance for the normal functioning of the ovary [6]. RIIβ is hormonally up-regulated during follicular development with expression of RIIβ reaching a peak level at a highly differentiated state of follicle development in response to a luteinizing hormone (LH) surge and elevated intracellular concentrations of cAMP. The importance of the sequence of these events led us to expect a high sensitivity of ovarian cells to any modulations of the cAMP- dependent pathway, despite possible deregulation of this system in an established ovarian cancer cell line. Moreover, previous studies had showed the possibility of ovarian cancer cell growth inhibition with the use of RIα antisense oligonucleotides [7].

## Methods

### Materials

OVCAR-8, human ovarian cancer cells, were obtained from DCT-Tumor Repository (NCI – Frederick Cancer Research Center). Tissue culture reagents were purchased from (Invitrogen, Inc., Carlsbad, CA). Monoclonal antibody for RIα, RIIα, and RIIβ were purchased from BD Biosciences Pharmingen (San Diego, CA). Polyclonal antibodies for RAB25 were kindly provided by Dr. K. Cheng from MD Anderson Cancer Center, University of Taxes. All other antibodies were purchased from Santa Cruz Biotechnology, Inc. (Santa Cruz, CA). DOTAP was purchased from Roche Applied Science (Indianapolis, IN). PKA inhibitor H89 and Protease Inhibitor Cocktail I were obtained from EMD Biosciences (Darmstadt, Germany).

### Cell culture and treatment

OVCAR-8 cells were grown in RPMI Medium 1640 supplemented with 10% heat-inactivated fetal bovine serum, MEM non-essential amino acids, and antibiotic-antimycotic, in a humidified incubator (95% air and 5% CO_2_) at 37°C. The stable transfectants, containing retroviral vectors OT1521 or OT1529 with the internal inducible mouse metallothionine-1 (MT-1) promoter, and genes encoding PKA subunits RIα and RIIβ were obtained as previously described [8]. For maximal induction of PKA genes without cytotoxicity, cells were treated with 60 uM ZnSO_4 _for 6 days prior to the start of the experiment. To exclude other effects of Zn++, parental cells were treated with ZnSO_4 _in the same manner as transfected cells.

### Cell morphology studies

Whole-cell morphology was determined as previously described [9]. Briefly, cells were washed with PBS, fixed with 70% methanol and stained with Giemsa (BioRad, Hercules, CA) according to the manufacturer's instructions. After staining, the cells were viewed under the microscope.

### Western blotting

Western blot analysis of proteins in parental and transfected OVCAR-8 cells was performed as described earlier [9]. Briefly, cells were lysed by homogenization in 20 mM Tris-HCl, pH 7.5, 100 mM NaCl, 5 mM MgCl_2_, 1% NP40, 0.5% sodium deoxycholate, Protease Inhibitor Cocktail I with subsequent centrifugation at 10 000 rpm, 10 min, 4°C. Equal amounts of protein lysate were subjected to SDS-PAGE, transferred to nitrocellulose membranes, and probed with antibodies as indicated in figure legends. Complexes were visualized with the appropriate horseradish peroxidase-conjugated secondary antibody and developed by enhanced chemiluminescence procedure (Santa Cruz Biotechnology, Santa Cruz, CA).

### RNA purification, northern blot analysis, microarray probe labeling

Total RNA was extracted using the Trizol Reagent method (Invitrogen, Carlsbad, California 92008, cat. no. 15596-026). Northern blot analysis was performed as described earlier [8]. Additional purification was performed on RNAeasy columns (Qiagen, Valencia, CA 913555, cat. no. 74104). The quality of total RNA samples was assessed using an Agilent 2100 Bioanalyzer (Agilent Technologies, Palo Alto, CA).

RNA samples were labeled according to the manufacturers recommended protocols. In short, 0.5 μg of total RNA from each sample was labeled by using the Illumina RNA Amplification Kit (Ambion, Austin, TX 78744-1832, cat. no. I1755). Single stranded RNA (cRNA) was generated and labeled by incorporating biotin-16-UTP (Roche Diagnostics GmbH, Mannheim, Germany, cat. no. 11388908910). 0.85 ugs of biotin-labeled cRNA was hybridized (16 hours) to Illumina's Sentrix HumanRef-8 Expression BeadChips (Illumina, San Diego, CA 92121-1975, cat.no. 11201828). The hybridized biotinylated cRNA was detected with streptavidin-Cy3 and quantitated using Illumina's BeadStation 500GX Genetic Analysis Systems scanner.

For Affymetrix, 5 μg of total RNA from each sample was converted into double-stranded cDNA by using the SuperScript Choice system (Invitrogen) with an oligo-dT primer containing the T7 RNA polymerase promoter. The double-stranded cDNA was used in an *in vitro* transcription reaction using the BioArray RNA transcript labeling kit (Enzo Life Sciences, Inc., Farmingdale, NY 11735). 20 ugs of biotin-labeled cRNA was fragmented and hybridized (16 hours) to Affymetrix GeneChip^® ^Human Genome U133 Plus 2.0 Arrays. The hybridized biotinylated cRNA was detected with phycoerythrin-streptavidin and quantitated by scanning (Affymetrix GeneChip Scanner).

### Quantitative RT-PCR (QRT-PCR) analysis

Reverse transcription was performed using total RNA isolated from tissue or cells and processed with Applied Biosystems (Foster City, CA) High-Capacity cDNA Archive kit first-strand synthesis system for RT-PCR according to the manufacturer's protocol. QRT-PCR was performed using the TaqMan assay system from Applied Biosystems. All PCR amplifications were carried out in duplicate on an ABI Prism^® ^7300 Sequence Detection System, using a fluorogenic 5' nuclease assay (TaqMan^® ^probes). Probes and primers were designed and synthesized by Applied Biosystems. Relative gene expressions are calculated by using the 2^-ΔΔCt ^method, in which Ct indicates cycle threshold, the fractional cycle number where the fluorescent signal reaches detection threshold [10]. The normalized ΔCt value of each sample is calculated using a total of 3 endogenous control genes (gapdh, actb, and pgk1). Fold change values are presented as average fold change = 2^-(averageΔΔCt) ^for genes in treated relative to control samples. Error bars represent the SEM for multiple biological replicates.

### Array data analysis

Preliminary analysis of Illumina data was performed using Illumina BeadStudio software which returns the trimmed mean average intensity for each single gene probe type (non-normalized). Any gene consistently below a background threshold level of D = .98 for all samples was eliminated from further analysis. This background filter resulted in the removal of approximately 55% of all the genes on the Illumina array. Z-transformation for normalization was performed on the remaining 11,048 genes for each Illumina sample/array on a stand-alone basis [11] [see Additional File 1].

Affymetrix data in the form of CEL files was normalized by the RMA method [12,13] using the web-based Bioconductor software [12,14]. Probes close to or at background were eliminated if any probe stayed consistently in the lower (average) quartile for all assayed samples.

Significant changes in gene expression were calculated for either RIA or RIIB versus control by Z test [15,16]. In addition, the mean difference of all calculated changes in gene expression was expressed in units of standard deviation from the average change of all genes for that comparison and referred to as a Z ratio. Z ratios are a direct measure of the likelihood that an observed change is an outlier in an otherwise normal distribution and their use allows for the detection of large changes with high variance which might otherwise be missed by conventional significance testing. Significant genelists were calculated for all categories (RIA or RIIB, up or down, separately for both Affymetrix and Illumina data) by selecting genes which satisfied significance threshold criteria of Z test p- values less than or equal to 0.001 (10^-3^), a false discovery rate less than or equal to 0.1 [17], and a fold change ± 2 or greater, or a Z ratio value greater than ± 3.0 [see Additional File 2]. Proliferation/differentiation genes were computed by the simple criteria that their average gene expression was either RIα > Control > RIIβ (proliferation) or RIα < Control < RIIβ (differentiation) [see Additional File 3].

In order to combine data from both Affymetrix and the Illumina gene expression arrays, we first mapped the gene annotations for each platform to the Human Genome Organization (HUGO) human gene symbols and names. Only genes with a positive identification to a HUGO gene were retained and this list was trimmed further to remove genes consistently at or below background as measured for either platform (as described above). Finally, duplicate gene probes were averaged leaving a core group of 7,170 genes common to both platforms, identified and used in subsequent analyses.

The myotube formation dataset was derived from a total of 48 samples corresponding to a time course of nine days of cells in culture following serum withdrawal (John Hall, personal communication). A minimum of 5 replicates at each time point were tested. In addition, cells were tested with and without gamma interferon as an internal control. A total of 8,446 genes were identified as being present and which had a clear functional annotation and were retained for further analysis. Significant changes in gene expression were calculated by the criteria outlined above by either comparing the average gene expression of the entire time course (day 1–day 9) or the interferon treated cells to the average gene expression at baseline.

Hierarchical clustering was performed using the Cluster and TreeView software programs, developed at Stanford University [18]. The clustering algorithm was set to complete linkage clustering using the uncentered Pearson correlation.

### GSMA, GSEA, and functional annotation

Gene Set Matrix Analysis (GSMA) [19] was performed using the median differences for differentially expressed genes tested against genesets derived from a variety of sources (for example, the Pathway genelists were originally obtained from the Gene Set Enrichment Analysis (GSEA) website maintained by the Broad Institute @ .

PAGE calculations [20] were automatically derived using a custom script for the GSMA algorithm implemented in JMP (v6.0) the statistical analysis software from the SAS Institute (Cary, NC), according to the formula:

Z = (Sm - μ)*m^1/2^/σ

where **Sm **is the median of Z ratio values of genes for a given gene set and the size of the given gene set is **m**. The median of total Z ratio values (**μ**) and standard deviation of total Z ratio values (**σ**) of a given microarray data set were calculated for all genes between two experimental groups.

A variation of this procedure is the substitution of different gene expression change measures such as fold changes, logratios, or p-values, so, for example, both z ratios and the log of fold changes were independently tested in GSMA for both Affymetrix and Illumina measurements [see Additional File 4]. These measurements were shown to give highly concordant results at the gene set level.

Z ratios were used as the gene expression change metric to test the OVCAR significantly regulated genelists by GSMA against the myoblast-myotube dataset. The top 250 genes (by z ratio) of each significant genelist for either RIα or RIIβ regulated genes were used for both GSMA and GSEA analysis.

Functional annotation was performed using the Database for Annotation, Visualization and Integrated Discovery (DAVID), NIAID/NIH [21,22].

## Results

### Overexpression of PKA subunits

**Figure 1:**
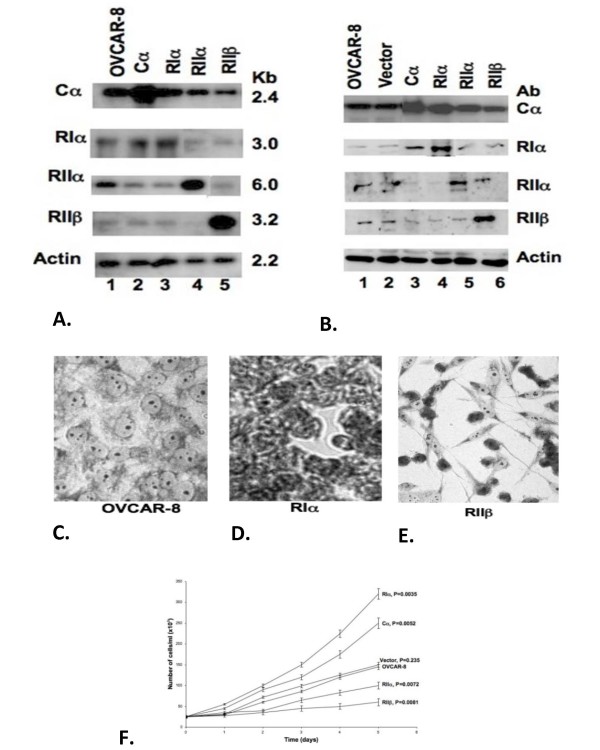
**PKA subunit overexpression in human ovarian cancer cells (OVCAR8).** 1A. Northern blot analysis of transfectants: parental cells, vector only, Cα PKA catalytic subunit, RIα, RIIα, RIIβ regulatory subunits as indicated. 1B. Western blot analysis – same transfectants. 1C. OVCAR 8 parental cells in culture. 1D. RIα-transfected cells in culture. 1E. RIIβ-transfected cells in culture. 1F. Growth response curves for PKA subunit transfectants.

To study the role of PKA isozymes in the regulation of cell proliferation *in vitro*, OVCAR-8 human ovarian cancer cells were transduced with retroviral vectors containing human PKA subunit genes, including the coding sequences for RIα, RIIα, RIIβ, and the catalytic subunit Cα. As shown in Figure 1A, a marked increase was seen for the mRNA levels of PKA subunits in transfectants, while in cells receiving the control vector, the subunit mRNA levels remained unchanged compared with the parental cells. Similarly, Western blot analysis (as shown in Figure 1B) confirmed the specific overexpression of PKA subunit proteins as compared with the parental cells.

We next examined the growth properties of the PKA subunit gene transfectants *in vitro* and determined that in monolayer culture, as expected, RIIβ transfectants showed a dramatic growth inhibition while, in contrast, RIα transfectants grew at a much faster rate than parental control cells (Figure 1C–F). These results were not surprising in light of the fact that it had been previously demonstrated that blockade of PKA-I protein with RIα antisense oligonucleotides causes arrest of tumor cell growth, induces apoptosis, inhibits tyrosine kinase signaling, and blocks changes in cell morphology [7,23-27] while, conversely, preferential expression of PKA-II is found in normal non-proliferating tissues as well as in growth-arrested cells [28,29].

RIα and RIIβ PKA subunit transfected cells were chosen for further characterization based upon their distinct and diametrically opposed phenotypes relative to each other and to their parental (OVCAR) cell line. The cellular morphology of RIα, parental, and RIIβ cells appeared to define an axis which in the parental to RIα direction leads to uncontrolled cell growth and proliferation and in the parental to RIIβ direction leads to apparent cellular differentiation and quiescence. We set out to determine whether this axis could be defined at the molecular level, on the basis of changes in global gene expression.

### Microarray analysis of subunit gene expression

**Figure 2:**
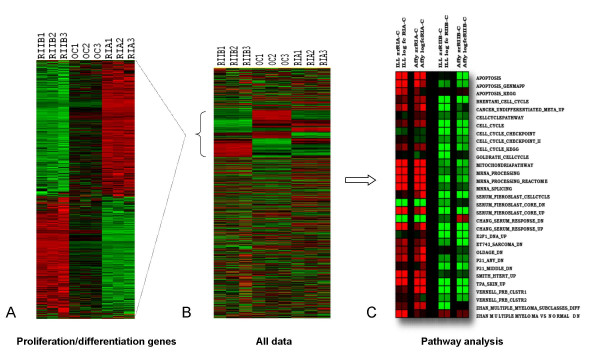
**Heatmap visualization of gene expression data from PKA subunit transfections.** 2A. Combined proliferation/differentiation genes (1933/1660 genes, respectively: Supplementary Data 4) computationally derived from a total of 11,048 genes detected (2B, and Supplementary Data 1). All samples in triplicate (biological replicates), gene expression normalized by rows as well as by individual samples to enhance visualization. 2C. Heatmap of a subset of GSMA enrichment scores using gene lists of pathway and gene signatures from the Broad Institute (MSigDB c2: Curated Gene Sets) (see Supplementary Data 3 for all GSMA scores). Red indicates positive enrichment (median gene expression of all genes in list > 0), green indicates negative enrichment (median gene expression of all genes in list < 0). Datasets tested include z ratios and log fold changes generated for PKA subunits samples using both Affymetrix U133 Plus 2.0 arrays as well as Illumina BeadArrays.

**Figure 3:**
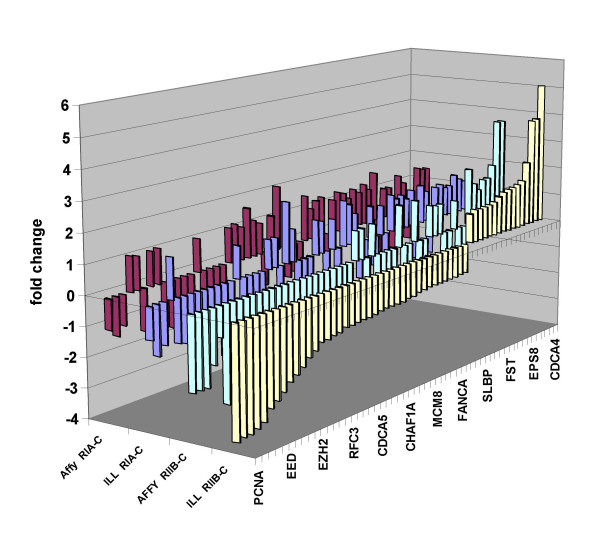
**Graphical representation of a subset of genes taken from one of the genelists tested by GSMA ((Vernell_PRB_Clstr2) and found to be negatively enriched for down-regulated genes in RIIβ transfected cells but not in RIA transfected cells, each relative to parental cells.** Fold changes (fc) derived from either the Affymetrix or Illumina platforms are shown. Featured genes include those involved in cell division.

Three biological replicates for each of the two subunit cell lines (RIα and RIIβ) and the parental OVCAR cells were used for microarray measurement analysis using several different platforms including Illumina, Agilent, and Affymetrix [30]. The overall gene expression results were shown to be highly correlated between platforms as illustrated, for example, by the similar results in calculations of selected regulated pathways obtained using either Affymetrix or Illumina (Figure 2C, Figure 3). For the purposes of this report, only the microarray measurements generated using Illumina BeadArrays will be used for all data analysis and results unless otherwise specifically indicated. A total of 11,048 genes were called present out of 24,350 genes tested on the arrays and were used as the basis group for further analysis. All present genes are displayed in the heat map in Figure 2B constructed by processing the data using unsupervised hierarchical clustering [18]. In this view, only the gene expression values have been re-sorted and the sample order has been held constant in order to visibly demonstrate both the reproducibility of replicates (r^2 ^> 0.99) [30] within samples as well as the dramatic changes in gene expression by sample type. A subset of the results from a preliminary pathway analysis of all the data by Gene Set Matrix Analysis (GSMA) [19] focusing on the global regulation of either RIα or RIIβ versus parental is displayed in Figure 2C. Multiple pathways are differentially regulated between RIα and RIIβ cell types including genes related to apoptosis, energy metabolism (mitochondrial pathway), mRNA processing and splicing, and cell cycle. Interestingly, regulated genes of the RIα and RIIβ cell types versus parental were shown to be concordant with results from a series of experiments in which fifty fibroblast cultures derived from ten anatomic sites were cultured in 10% fetal bovine serum and a stereotyped gene expression program identifying genes that were reproducibly induced or repressed in fibroblasts in response to serum was identified [31]. These genes (Fig. 2C, CHANG_SERUM_RESPONSE_UP or DN) were enriched in RIα and RIIβ cells for up and down-regulation matching serum stimulated (RIα) or serum depressed (RIIβ) gene expression. Furthermore, Chang et al. experimentally derived a subset of serum activated genes which eliminated the contributions of genes directly related to cell proliferation (i.e. directly related to cell cycle progression). Testing of RIα and RIIβ regulated genes against this core group of genes (SERUM_FIBROBLAST_CORE_UP or DN) gave similar results as before, demonstrating that the changes in gene expression induced by these two PKA subunits went well beyond just simple alterations of cell cycle. The genes induced in the fibroblast serum-response program are expressed in tumors, by the tumor cells themselves, and by tumor-associated fibroblasts. They are evident at an early clinical stage and predict increased risk of metastasis and death in breast, lung, and gastric carcinomas [31]. Further concordance of RIα and RIIβ regulated genes was demonstrated with other empirically derived gene expression signatures related to cancer and cancer progression, including, for example, the down-regulation of genes in RIIβ cells related to DNA replication (E2F1_DNA_UP) [32], as well as to genes down-regulated following successful treatment of sarcoma cell lines with Ecteinascidin 743 (ET743_SARCOMA_DN) [33], and to p53-dependent down-regulation of gene expression in the ovarian cancer cell line 2774 following the ectopic expression of p21(CDKN1A) (P21_P53_ANY or MIDDLE_DN) [34]. Conversely, the up-regulation of genes in RIα cells was shown to be concordant with genes up-regulated in mouse skin by the phorbol ester carcinogen, TPA (TPA_SKIN_UP) [35], with genes down regulated in human fibroblasts due to old-age and progeria (OLDAGE_DN) [36], with genes associated with the overexpression of human telomerase in human mammary epithelial cells (HMECs) leading to a diminished requirement for exogenous growth factors (SMITH_HTERT_UP) [37], and to 30 genes whose up-regulation most clearly defined the progression from MM1 to MM4 subgroups in patients with multiple myeloma, correlating to clinical parameters of poor prognosis for this cohort (ZHAN_MULTIPLE_MYELOMA_SUBCLASSES_DIFF) [38]. Figure 3 illustrates a breakout of one of these correlating gene expression signatures on a gene by gene basis (VERNELL_PRB_CLSTR1) which catalogues a group of genes which are up-regulated by E2F and down-regulated by pRB and p16 in human osteosarcoma cells. GSMA gene set analysis indicated that as a group, these genes were down regulated in RIIβ cells and, as shown in Fig. 3, include the specific down-regulation of genes involved in DNA replication and repair (PCNA, FANCA), and control of the cell cycle, and cell division (CDCA5).

### Identification and validation of key regulatory genes

**Figure 4:**
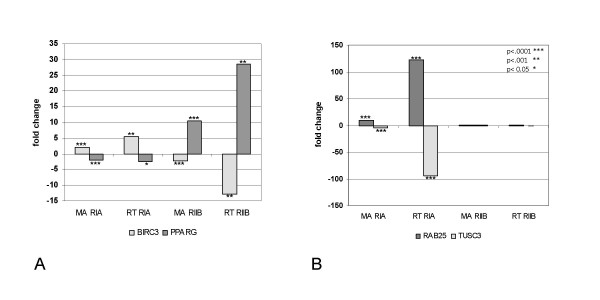
**RT-PCR validation of gene expression changes in selected PKA subunit proliferation/differentiation genes.** The fold changes for selected genes calculated from either RT-PCR (RT) or microarray (MA) are shown. T-test p-values are indicated by asterisks.

Several candidate genes identified in previous studies of ovarian cancer progression were identified in the PKA OVCAR model system by microarray and validated by RT-PCR (Figure 4). These genes include RAB25, a member of the RAS oncogene family, recently implicated in the regulation of cell proliferation and apoptosis in ovarian cancer cells [39] and with reports that tumor cells overexpressing the RAB25 protein were more aggressive and associated with a poorer clinical outcome [40]. A high level of RAB25 protein in patients with either breast or ovarian cancer was associated with an almost 50% reduction in five year survival rates. RAB25 mRNA levels were elevated between 10- (microarray) to 100-fold (RT-PCR), in RIα transfected OVCAR cells relative to either RIIβ transfected or parental OVCAR cells.

The loss of a tumor suppressor gene, tumor suppressor candidate 3 (TUSC3), was seen in RIα cells compared to either RIIβ transfected or to parental OVCAR cells. Loss of heterozygosity on chromosomal band 8p22 and decreased gene expression of TUSC3 has been associated with an increase of metastatic potential in prostate, colorectal, and ovarian cancer [41-43]. In particular, TUSC3 showed significantly lower expression in grade 3 primary ovarian carcinoma tumors compared with tumors of lower grade or compared with normal controls [41]. Taken together, the dramatic up-regulation of RAB25 and down-regulation of TUSC3 in RIα cells appears to faithfully mimic the activity profiles of proven markers of ovarian cancer clinical progression.

Several other interesting genes displayed direct counter-regulation between RIα and RIIβ cells, including the human baculoviral IAP repeat-containing 3 (BIRC3) gene mRNA. The BIRC3 gene codes for a protein whose function includes the antagonism of the activation of apoptosis-promoting ICE-like proteases. Elevated levels of BIRC3 have been shown by others to promote tumor cell survival [44,45], and the down-regulation of BIRC3 in RIIβ cells may indicate the release of a block to apoptosis which may account for, in part, for the decreased cell number in this cell type. Counter-regulation in the opposite direction was displayed by the peroxisome proliferator-activated receptor gamma (PPARG) gene, which was highly up-regulated in RIIβ cells and mildly down-regulated in RIα cells. PPARG is a gene which has been shown to play a pivotal role in the processes of cellular differentiation, adipogenesis, and several reports connect PPARG status with neoplastic processes suggesting that PPARG may act as a tumor suppressor for some tissues and in some cellular contexts [46,47].

### Cross-model validation

**Figure 5:**
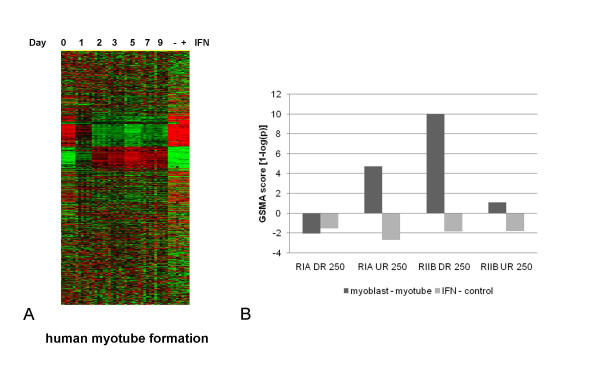
**Cross model validation of PKA subunit regulated genes versus human myotube differentiation genes.A**. Heat map of the hierarchical clustering of gene expression accompanying myotube induction following the withdrawal of serum from myoblasts in culture from baseline (day 0) to day 9. **B**. GSMA results using genelists corresponding to the top 250 RIα and RIIβ significantly up- or down-regulated genes versus either the myotube dataset or IFN-treated myoblasts (as a negative control). Significant concordance between RIα up-regulated and, conversely, RIIβ down-regulated genes with myoblast up-regulated genes is shown. GSMA scores are shown as significance values [1-log(p)] generated from a two-tailed test of the Standard Normal Distribution.

PKA regulated genes were tested directly against an unrelated *in vitro* model system of cellular proliferation/differentiation for confirmation that these data were representative of a generic cellular response. A gene expression profile for a time course of human myotube formation (Figure 5A) was used for comparison and will be described in detail elsewhere. Examination of the myotube gene expression profile shows that the major programmatic changes start almost immediately following serum withdrawal and remain relatively constant throughout the time course.

Genelists were derived from the OVCAR data using the top 250 genes (by size of fold change) significantly regulated for either RIα or RIIβ cells versus parental cells as previously described [30] and these genelists were tested against all calculated changes in gene expression between day 2–9 and day 0–1 from the myotube dataset for concordance by Gene Set Matrix Analysis (GSMA) analysis [19]. The results as shown in Figure 5B, demonstrated a highly significant correlation for RIα up-regulated genes ((p < 2E-4) and RIIβ down-regulated genes (p < 1E-11) and the gene expression differences which distinguish myoblasts from myotube cells. Genes up-regulated in rapidly dividing myocytes correlate well with genes up-regulated by RIα or down-regulated by RIIβ in OVCAR cells. These apparently unrelated, and certainly biologically distinct, cell types are connected by a common theme at the level of gene expression.

**Figure 6:**
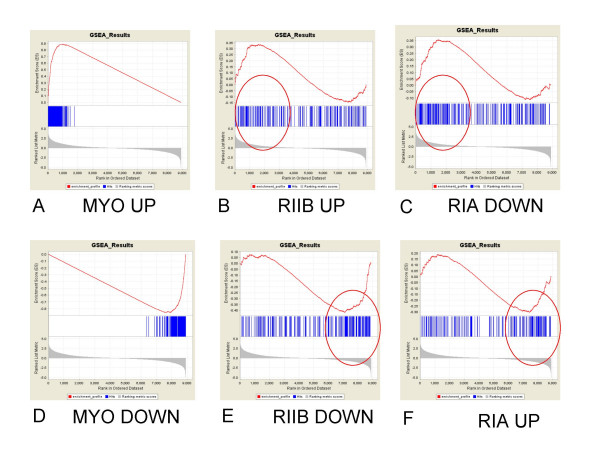
**GSEA gene set analysis of PKA subunit regulated genes versus human myotube differentiation data.** The top 250 RIα, RIIβ, and myotube significantly up- and down-regulated genes are graphically displayed as indicated across a rank ordered distribution of all myotube versus myoblast changes in gene expression. The red curves report the values of a modified Kolmogorov-Smirnoff cumulative statistic [50] across the distribution of all changes in gene expression (of the myotube dataset). The peak of the curve (either above or below the midpoint of zero) becomes the maximum enrichment score (ES) which is normalized by gene list size to generate a normalized enrichment score (NES) and tested for significance by permutation analysis (NOM p-val, as reported in Table 1). Myotube up- or down-regulated genelists were used as positive controls. RIIβ and RIα up- and down-regulated genes are shown to cross correlated with myotube changes in gene expression (red circles indicate areas of enrichment).

As a cross check to the GSMA calculations, we decided to test the same dataset/genelist combination using another increasingly popular gene set analysis method, Gene Set Enrichment Analysis (GSEA) [48-50]. In this instance, genelists derived from significantly regulated genes in the myotube/myoblast dataset were used as positive controls and OVCAR PKA subunit significant genelists derived from three different commercial platforms [51] were tested simultaneously against the entire myoblast/myotube dataset. GSEA, without supervision, grouped all the submitted genelists in the predicted direction (Table 1), i.e. RIIβ up-regulated and RIα down-regulated genelists were associated with myotube formation (MYO_UP), while RIα up-regulated and RIIβ down-regulated genelists were associated with myoblasts (MYO_DOWN). In GSEA, the difference data is rank ordered from the largest positive change to the largest negative change (the grey profile at the bottom of each GSEA graph – Figure 6). Genes from the submitted genelists are mapped to this distribution (blue bar code) and enrichment for any particular gene list at either end of the distribution is statistically calculated. The Illumina derived OVCAR gene lists, in particular, performed consistently well, with three of the four lists surviving a stringent permutation estimate of significance (NOM p-val) followed by a multiple comparison correction (FDR q-val < 0.1). The functional annotation of the core group of genes (highlighted in Fig. 6 by red circles) corresponding to the overlap between RIα and myoblast up-regulated genes included genes involved in energy metabolism, mRNA processing, and ribosome formation. Functional annotation of the core group of genes corresponding to the overlap between RIβ and myotube up-regulated genes included genes involved in the formation of extracellular matrix, focal adhesion and cell-cell communication. In the ovarian cancer cell model we have used, a shift in the relative amounts of the RIα and the RIIβ regulatory subunits of PKA is sufficient to trigger a programmatic shift that both morphologically and at the level of gene expression looks very much like a generic program of cellular proliferation and differentiation.

**Table 1:** GSEA gene set analysis of PKA subunit regulated genes versus human myotube differentiation data.

**NAME**	**SIZE**	**ES**	**NES**	**NOM p-val**	**FDR q-val**
**MYOTUBE_UPREG**	**250**	**0.889**	**1.607**	**0.007**	**0.005**
**RIIB_UPREG_OC_AFFY_TOP250**	**143**	**0.335**	**1.465**	**0.008**	**0.037**
**RIA_DOWNREG_OC_ILLUMINA_TOP250**	**225**	**0.357**	**1.368**	**0.03**	**0.057**
**RIIB_UPREG_OC_ILLUMINA_TOP250**	**199**	**0.337**	**1.363**	**0.037**	**0.048**
**RIA_DOWNREG_OC_AFFY_TOP250**	**157**	**0.317**	**1.221**	**0.14**	**0.145**
					
**MYOTUBE_DOWNREG**	**250**	**-0.857**	**-1.563**	**0.007**	**0.014**
**RIIB_DOWNREG_OC_ILLUMINA_TOP250**	**222**	**-0.418**	**-1.496**	**0.012**	**0.021**
RIA_UPREG_OC_ILLUMINA_TOP250	175	-0.305	-1.148	0.254	0.51
RIIB_DOWNREG_OC_AFFY_TOP250	178	-0.296	-1.038	0.369	0.451
RIA_UPREG_OC_AFFY_TOP250	153	-0.24	-0.932	0.591	0.563

The top 250 up- or down-regulated genes from either RIα or RIIβ cells were derived from independent measurements made on either Affymetrix or Illumina microarrays and tested against the results of a human myotube versus myoblast comparison dataset. Bolded rows indicate statically significant results (FDR q-val < 0.25). MYOTUBE_UP and MYOTUBE_DOWN are control genelists derived from the top 250 up- or down-regulated genes derived directly from the myotube dataset. Size refers to the number of genes in the dataset found using a particular gene list, ES = enrichment score, NES = normalized enrichment score, NOM = nominal p-value following permutation analysis, FDR = false discovery rate (multiple comparisons correction), FWER = family wise error rate. For a complete discussion of this method see GSEA Algorithm.

## Discussion

We chose to characterize RIα and RIIβ transfected ovarian cancer cells where the relative levels of Type I and Type II PKA isozymes have been deliberately shifted with obvious and profound consequences (Fig. 1C–E). It appeared likely that these effects would also be paralleled at the level of gene expression and they were indeed reproducible and profound (Fig. 2B). In order to directly investigate the apparent antagonistic effects of RIα and RIIβ in ovarian cancer cells at the gene expression level, a large group of genes was identified solely on the basis that their gene expression progressed through the RIα-parental-RIIβ axis and these genes were tested for biological relevancy by looking for patterns of functional enrichment of genes involved in proliferation (RIα > parental > RIIβ), or differentiation (RIα < parental < RIIβ). We performed gene set analysis on these data and showed that the association of RIα and RIIβ with proliferation/differentiation genes was consistent with the resulting patterns of GSMA enrichment scores, a subset of which is featured in Fig. 2C. One particularly striking example of this enrichment was the precise overlap of RIα and RIIβ with up- and down-regulation in the Chang serum response data [31]. The Chang study investigated aspects of cancer invasion and metastasis that appear to overlap with the gene expression pattern of wound healing (modeled by response to serum exposure) since genes induced in the fibroblast serum-response program are often found to be coordinately up-regulated in many human tumors as well [31]. Our data tends to support their hypothesis of a connection since RIα-induced gene expression not only correlates well with a positive response to serum but also simultaneously significantly up-regulates genes, like RAB25 (Fig. 4), independently associated with advanced metastasis in ovarian cell cancer.

Over-expression of RAB25 in both breast and ovarian cancer cells reportedly decreased apoptosis and increased proliferation of these cells in culture and increased their aggressiveness in vivo [52]. RAB25 was among a group of genes whose overexpression distinguished ovarian/primary peritoneal serous carcinoma (OC/PPC) from diffuse peritoneal malignant mesothelioma (DMPM), two highly aggressive tumor types which are closely related, both morphologically and histogenetically [53]. All evidence to date suggests that increased RAB25 gene expression (both by increases in DNA copy number as well as accompanying increases in mRNA expression levels [54]) is specifically associated with ovarian cancer progression and our results demonstrate that elevated levels of the RIα PKA regulatory subunit can lead directly to elevated levels of RAB25 in ovarian cancer cells. Similarly, the TUSC gene encodes a protein with oligosaccahryl transferase activity which was originally identified as a homozygous deletion in metastatic prostate cancer [42]. TUSC3 has since been shown to be hypermethylated in acute lymphoblastoid leukemia (ALL) cells [55], as well as in cervical intraepitelial neoplasia [56], and to be specifically associated with loss of heterozygosity (LOH) on chromosomal band 8p22, a common event in several epithelial tumors including ovarian carcinoma [41]. Significantly lower expression of TUSC3 was correlated with an increase in clinical grade severity in a study of 58 primary ovarian carcinoma tissues [41]. The overexpression of RIα PKA regulatory subunit leads directly to significantly decreased levels of TUSC3 in the OVCAR PKA subunit model system.

We have accumulated evidence from cell growth and morphology, from pathway involvement in global gene expression patterns, and from key regulatory genes which act as biomarkers for clinical ovarian cancer progression, that the transfection of PKA regulatory subunits in an ovarian cancer cell line directly controls cell fate along an axis which we have characterized as proliferation/differentiation (RIα-parental-RIIβ). We tested this characterization directly by correlating significant changes in gene expression derived from the OVCAR model system against the entire dataset of gene expression changes generated from an *in vitro* model system of human myotube formation using both a parametric (GSMA) and a non-parametric (GSEA) approach. Both analytical techniques returned results, without supervision, which indicated a significant overlap between the two model systems.

While it is always hazardous to extrapolate without hesitation from immortalized cell lines in tissue culture into the behavior of cells in an intact environment under various physiological conditions and stresses, cell lines, however, are still widely used as *in vitro* models in cancer research because they are relatively easy to handle, and are a renewable resource that can be grown in almost infinite quantities [57]. In general, they exhibit a relatively high degree of homogeneity and preserve *in vitro*, the genetic aberrations unique to their parent histology from which they were derived despite the fact that they are prone to a measurable level of genotypic and phenotypic drift during their continual culture [58]. Despite these caveats we have demonstrated that an established ovarian epithelial cancel line, OVCAR8, can be manipulated by the selective overexpression of PKA regulatory subunits into states, which at the level of gene expression, are clearly associated with either progression or differentiation. We show that these states are both general (canonical pathway regulation) as well as specifically correlated with patterns of gene expression related to cancer progression as reported from other publicly available microarray studies. We provide evidence on a gene-by-gene basis that biomarkers specifically associated with poor prognosis in ovarian cancer (elevated levels of RAB25, decreased levels of TUSC3) are dramatically elevated by RIα over expression in a cell line which may well be acting, in this case, as a stage-specific surrogate to a full blown ovarian cancer cell malignancy. Finally, we provide evidence that this stage-specific transition can not only be triggered solely by the differential expression of PKA regulatory subunits but also correlates well with an, otherwise, unrelated model system of proliferation/differentiation generated by the mapping of gene expression changes between myoblast and myotube cells.

## Conclusion

The overexpression of PKA regulatory subunits in an ovarian cancer cell model has been used to show that cells enter either a hyperproliferative (RIα) or a quasi-quiescent (RIIβ) phenotypic state which, at the level of gene expression, mimics changes in gene expression associated with good or poor prognosis in ovarian cancer clinical outcomes. These patterns of gene expression are also clearly related to a more generalized pattern of gene expression in other model systems of cellular proliferation and differentiation. Taken altogether, these observations support the conclusion, already suggested by a great deal of previous work, that whether causative or in part as a reaction to other cellular events, the state and condition of PKA remains a fundamental determinant of cell fate.

## Abbreviations

Riα: RI alpha; RIIβ: RII beta; GSEA: Gene Set Enrichment Analysis; GSMA: Gene Set Matrix Analysis; PAGE: Parametric Analysis of Gene Enrichment.

## Competing interests

The authors declare that they have no competing interests.

## Authors' contributions

MN performed the cell culture, transfections, Western and Northern blots. CC and TW performed the microarray assays. CC performed the QRT-PCR, carried out the statistical analysis, and drafted the manuscript. JCH and AR contributed the myotube data. KCB and YSC-C participated in the design of the study. All authors read and approved the final manuscript.

## Pre-publication history

The pre-publication history for this paper can be accessed here:



## Supplementary Material

Additional file 1 **Data** Click here for file

Additional file 2 **Table** Click here for file

Additional file 3 **Table** Click here for file

Additional file 4 **Table** Click here for file
